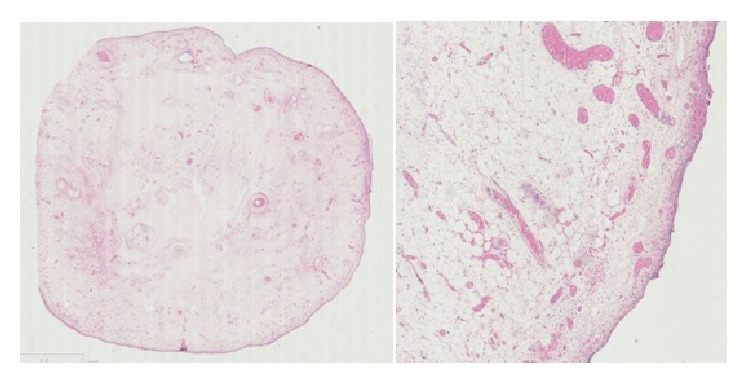# Lipoma of Piriform Sinus: A Case Report and Review of the Literature

**DOI:** 10.1155/2016/2521583

**Published:** 2016-10-04

**Authors:** Gilberto Acquaviva, Theodoros Varakliotis, Stefano Badia, Francesco Casorati, Alberto Eibenstein, Gianluca Bellocchi

**Affiliations:** ^1^Department of Otolaryngology and Head & Neck Surgery, “San Camillo-Forlanini” Hospital, Rome, Italy; ^2^Department of Applied Clinical Sciences and Biotechnology (DISCAB), L'Aquila University, L'Aquila, Italy

## Abstract

The lipomas of oropharynx, hypopharynx, and larynx are so rare that up to now approximately there have been 100 cases reported. The lipomas are slow-growing lesions that are capable of reaching considerable dimensions and are often detected at a late stage. The symptoms can vary both in dimension and in location, semiobstructing the aerodigestive tract or exerting compression on adjacent structure. In this case, the lesion, which originated from the piriform sinus, was removed endoscopically urgently due to obvious signs of tissue suffering caused by stretching of the pedicle as a result of displacement of the mass. The two aims of this case report are to expose an interesting and rare case study mainly for an Emergency Room Specialist and an ENT (Ear, Nose, and Throat) Specialist involved in solving the problem and to demonstrate that the choice of an endoscopic approach is useful in order to have an optimal visualization of the lesion and to perform a total eradication. The use of endoscopic devices also allows a rapid postoperative recovery, compared to external access and optimum locoregional control in the follow-up procedures to prevent possible relapses.

## 1. Introduction

Lipomas are tumors that represent 4-5% of all benign tumors and they are predominantly found in the hypodermic layer [1].

These tumors are rare in the head and neck region and specifically for the upper aerodigestive tract represent 0.6% of all neoplasias [2, 3].

We present a rare case of a 63-year-old woman who arrived at the emergency room of our hospital with a pedunculated mass protruding from the buccal rhyme, emitted by coughing.

## 2. Case Report

**Figure 1:**
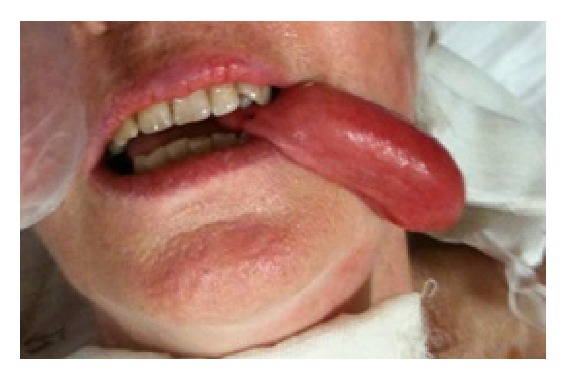


In August of 2011 a 63-year-old woman suffering from multiple sclerosis and diabetes mellitus type I, in treatment with insulin since the age of 18, arrived at the emergency room with severe dyspneic syndrome. She presented a voluminous mass, partially protruding from the mouth and completely occupying the oral cavity, looking diverticular with a solid consistency and brownish-yellow color, cylindrical, and smooth, with a diameter of about 25 mm. The protruding portion from the mouth measured in length about 65 mm (Figure 1).

The relatives, who accompanied the patient, reported a sudden onset of dyspnea and the appearance from the mouth of the abovementioned lesion protruding after regurgitation and coughing. After the first examination it was clear that the tumor extended in the hypopharynx with slightly smaller dimensions. The patient was immediately submitted to an endotracheal intubation, and an urgent CT scan of the neck was carried out to identify the relationship and the cause of the neoformation.

**Figure 2:**
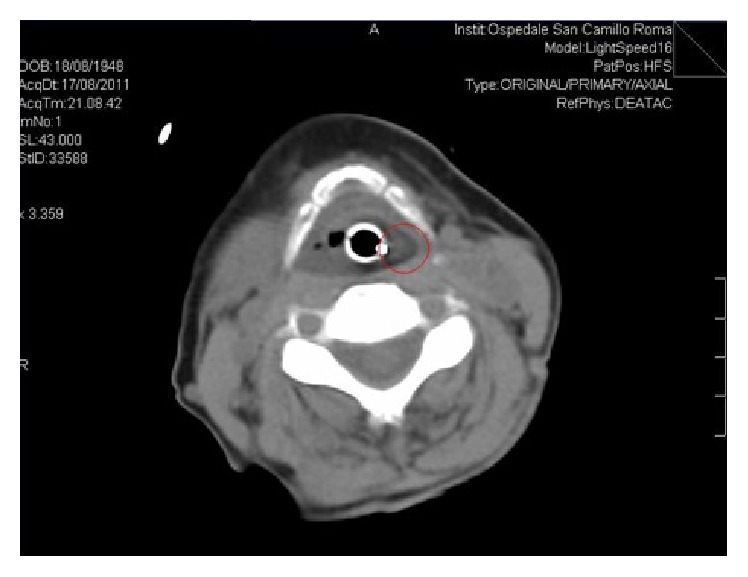


The CT scan showed the presence of solid polypoid-like tissue that occupied the space from the hypopharynx to the oral cavity with a large extraoral hypodense component having thin branches of vascularity to the left of the center section; the first hypothesis could be defined as a fibrovascular polyp with the implantation on the left side of the hypopharynx (Figure 2).

**Figure 3:**
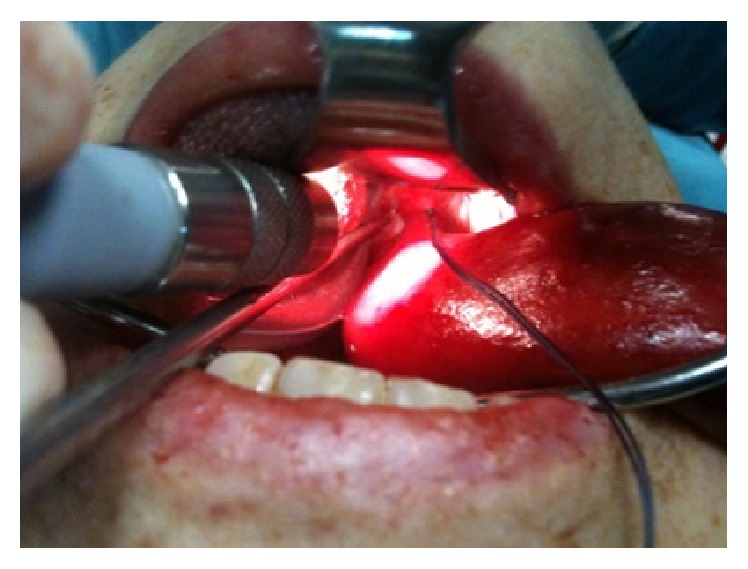


The microlaryngoscopy exploration, performed immediately due to obvious signs of the insufficient blood supply of the neoformation with the risk of ulceration and consequent bleeding, showed instead that the base of implant, also cylindrical, but with a diameter of about 10 mm, was located in area of the medial wall of the piriform sinus on the right (Figure 3).

**Figure 4:**
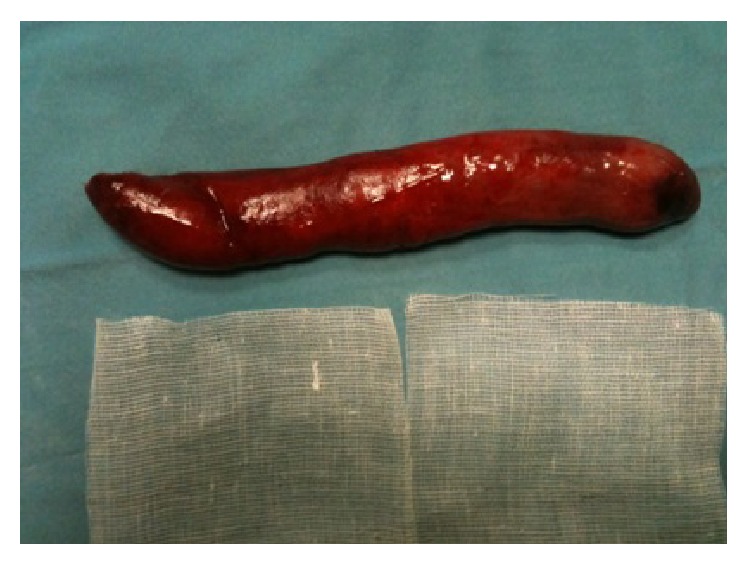


During the procedure, we used a Polysorb n.1 lace as a landmark and as point of traction at the base of implantation of the neoformation which was subsequently sutured with 2 stitches of Monocryl 2-0 and 2 stitches of Polysorb 2-0. With the aid of mechanical stapler Endo Gia Universal 12 mm used to dissect the neoformation at the base, it was consequently removed and the material was sent for a definitive histological examination (Figure 4); then a nasogastric tube was inserted ensuring its position towards the left piriform sinus.

The final histological examination described an oval shape lesion with a size of 15 × 2,5 × 2 cm, smooth in texture, soft in consistency, and yellowish in color. Fifteen blocks in paraffin, one centimeter in diameter, were obtained and were available for microscopic examination. Microscopic examination demonstrated a subepithelial neoplasm not encapsulated, composed of mature adipocytes with no cellular atypia. The diagnosis of lipoma was thus proposed (Figure 5).

The postoperative recovery period was quick and without complications, with resumption of oral feeding after 72 hours. Upon the patient's discharge from the hospital the examination with fibrolaryngoscope showed the absence of residual disease and the advanced state of reepithelialization of the implant site.

## 3. Discussion

Lipomas are benign tumors of mesenchymal originating from mature fat cells (adipocytes) in adipose component tissue. The adipose tissue in the larynx is represented in epiglottis, in the ventricular and aryepiglottic fold.

In this case the origin of the adipose tissue was located in the lateral submucosa to the arytenoid cartilage.

Lipomas generally are slow-growing sessile or pedunculated lesions having a rough surface, yellowish color, and soft consistency.

Histologically these lesions can be classified into several subtype groups based on their stroma: angiomyolipoma, spindle cell lipomas, myolipoma, chondrolipoma, and myxolipoma [4].

However among the possible malignant histotypes we find liposarcoma having well-differentiated cells [5].

The microscopic histological characteristics that allow differentiating benign from malignant neoformation are the following: presence of pleomorphism, atypical cells, and infiltration of surrounding tissues and lipoblasts.

The suspicion of malignancy growth can be defined in the case of the presence of one or more relapses after surgical removal [6]; however the malignant transformation is rare in cases of single lipoma, considering that an association to multiple pharyngolaryngeal lipomas or relapsing lipomas has been described.

The etiology of lipomas is unknown; however some authors have suggested that they derive from lipoblasts or by a metaplasia of muscle cells, while others have suggested a possible etiopathological role of familiar and endocrine factors or conditions such as trauma, infection, or chronic diseases. Nowadays some risk factors such as abuse of alcohol, tobacco, and environmental and work conditions, which involve toxic chemicals, have not been recognized as a possible cause [1, 6].

Clinically the lipomas remain asymptomatic for a long time, but once they reach large dimensions, symptoms may arise in relation to the size and lead to the compression of adjacent structures [7].

Lipomas of the upper aerodigestive tract occur with continuous or intermittent symptoms over the course of a few months or years, which include dysphagia, hoarseness, feeling of discomfort in the throat like the feeling of a mass in the pharynx, and, in case of airway obstruction, stridor and dyspnea [8]. In severe but rare cases it is necessary to perform an emergency tracheostomy to prevent asphyxia and possible episodes of respiratory arrest [9].

The diagnosis of these lesions starts with a local physical examination (lipomas may appear as sessile or pedunculated lesions or as a retention cyst with a rough surface, encapsulated and covered by a yellowish-pink mucosa) [6]; and consequently, using endoscopic techniques (fibrolaryngoscopy and esophagoscopy), a lipoma may appear as a submucosal mass or a polyp sometimes pedunculated.

Imaging techniques such as CT and MRI are helpful for the diagnosis and allow an acceptable preoperative evaluation of the lesion. On CT images lipoma appears as a homogeneous lesion with low attenuation values of ionizing radiation with a density lower than water [10, 11]. The accuracy rate of CT ranges from 75% to 90%.

MRI is preferable to CT because it allows a better study of the soft tissues, the patient is not exposed to ionizing radiation, and it does not require iodinated radiocontrast agents. Furthermore MRI also allows a more accurate and specific diagnosis of the lesion with a better indication of position of the peduncle or of the extension of the lesion and its relationship with the surrounding structures [12].

In this case CT scan, performed in urgency, helped to identify the caudal limit of the lesion and therefore identifying its place of origin, but not showing its position. The choice of using an endoscopic access [13], despite the difficulties arising due to small dimensions of the surgical field, caused by the tumor mass, rather than a lateral pharyngotomy [14], has been proved correct for best framing and also effective for the purposes of treatment [15].

## 4. Conclusions

The lipomas are benign tumors, but because of their rare location found in the upper aerodigestive tract, they must be taken seriously by the Emergency Room Specialist, the ENT Specialists, and Maxillofacial Surgeons. In fact often these lesions are very voluminous and their rapid displacement from the area, in which they have been growing for a long time, may endanger the life of the patient due to obstruction of upper airways. After the stabilization of the patient, ensuring good and safe ventilation, it is necessary to proceed with a quick imaging study and then to perform an endoscopy exam in order to plan a correct treatment. When the endoscopic approach is technically possible, with ablation of the neoformation at the base of the root, it is possible to apply an effective treatment and to obtain a rapid postoperative recovery period.

## Figures and Tables

**Figure 5:**